# Abnormally Increased Secretion in Olfactory Neuronal Precursors from a Case of Schizophrenia Is Modulated by Melatonin: A Pilot Study

**DOI:** 10.3390/ijms18071439

**Published:** 2017-07-13

**Authors:** Montserrat G. Cercós, Tania Galván-Arrieta, Marcela Valdés-Tovar, Héctor Solís-Chagoyán, Jesús Argueta, Gloria Benítez-King, Citlali Trueta

**Affiliations:** 1 Departamento de Neurofisiología, Dirección de Investigaciones en Neurociencias, Instituto Nacional de Psiquiatría Ramón de la Fuente Muñiz, Calzada México-Xochimilco 101 San Lorenzo Huipulco, Tlalpan, Mexico City 14370, Mexico; montse@imp.edu.mx; 2 Laboratorio de Neurofarmacología, Subdirección de Investigaciones Clínicas, Instituto Nacional de Psiquiatría Ramón de la Fuente Muñiz, Calzada México-Xochimilco 101 San Lorenzo Huipulco, Tlalpan, Mexico City 14370, Mexico; mayahuel84@hotmail.com (T.G.-A.); mvaldes@imp.edu.mx (M.V.-T.); hecsolch@imp.edu.mx (H.S.-C.); jadclear@yahoo.com (J.A.); bekin@imp.edu.mx (G.B.-K.)

**Keywords:** schizophrenia, secretion/exocytosis, olfactory neuronal precursors, melatonin, actin cytoskeleton, secretory vesicles

## Abstract

The alterations that underlie the pathophysiology of schizophrenia (SCZ) include the dysregulation of structural and functional properties of neurons. Among these, the secretion of neurotransmitters and hormones, which plays a key role for neuronal communication and development, is altered. Neuronal precursors from the human olfactory epithelium have been recently characterized as a reliable model for studying the etiopathogenesis of neuropsychiatric diseases. Our previous work has shown that melatonin enhances the development of morphological and functional features of cloned olfactory neuronal precursors (ONPs) from a healthy subject. In this work we found that primary cultures of ONPs obtained from a schizophrenic patient display an increased potassium-evoked secretion, when compared with ONPs from an age- and gender-matched healthy control subject (HCS). Secretion was evaluated by FM1-43 fluorescence cumulative changes in response to depolarization. Interestingly, a 12 h-melatonin treatment modulated the abnormally increased secretion in SCZ ONPs and brought it to levels similar to those found in the HCS ONPs. Our results suggest that the actin cytoskeleton might be a target for melatonin effects, since it induces the thickening of actin microfilament bundles. Further research will address the mechanisms by which melatonin modulates neurochemical secretion from ONPs.

## 1. Introduction

Schizophrenia (SCZ) is a chronic neuropsychiatric disorder that has a significant impact on individuals and their families, leading to poor functional outcomes across multiple domains, including employment, living independence, and social functioning [1]. The lifetime prevalence of this disorder varies geographically, ranging from 0.4% to 1% in the general population, and typically manifests in late adolescence or early adulthood [2,3]. The disorder is characterized by positive symptoms (such as delusions, hallucinations, and disorganized speech), negative symptoms (such as blunted affect, alogia, anhedonia, asociality, and avolition), and cognitive impairment [4]. These symptoms reflect impairments that could be related to both structural and functional brain alterations in these patients. In schizophrenia, various studies have suggested a dysregulation of neurotransmission, in particular of the dopaminergic, glutamatergic, and GABAergic systems [5,6,7]. Among these alterations, brain imaging studies have documented increased activity of the dopaminergic system, which suggests that dopamine secretion might be exacerbated in the striatum of the patients [8,9,10]. An elevated amount of dopamine would bind to its D2-receptors, causing hyperactivation of the system (which has been found to correlate with the occurrence or worsening of the positive psychotic symptoms in at least 40% of patients). Dopaminergic hyperactivation is present at the onset of the disease and could also be associated with the relapse phases of the illness [10]. In agreement with these studies, Hook et al. recently reported that neurons derived from human induced pluripotent stem cells (hiPSC) of schizophrenic patients display an increased activity-dependent catecholamine secretion [11].

Despite this information, little is known about secretion in the nervous system of patients with SCZ. Secretion of neurotransmitters and neuropeptides in the synapses mediates the effective transmission of information in neural circuits. In addition, secretion at extrasynaptic sites mediates most of the paracrine communication between neurons and with glial and vascular cells, for an overall regulation of the nervous system function [12]. In both synaptic and extrasynaptic secretion sites, biochemical mediators are released to the extracellular milieu by exocytosis from secretory vesicles or granules, through their fusion with the plasma membrane. Because vesicles need to be transported from the Golgi apparatus to the release sites at the plasma membrane, exocytosis is modulated by several factors, among which the cytoskeleton plays a key role. In particular, the actin cytoskeleton has a double action on the secretory vesicles, depending on the activation status of the cell. On the one hand, while the cells are at rest, actin prevents secretory vesicles from reaching the plasma membrane; on the other hand, upon stimuli that trigger exocytosis, it provides tracks to direct the vesicles towards their release sites and participates in the fusion of vesicles and the extrusion of the secreted molecules, as well as in the endocytosis process by which the vesicular membrane is retrieved [13].

Actin cytoskeletal organization is modulated by growth factors and hormones. Among these, melatonin (*N*-acetyl-5-methoxytryptamine)—the main indoleamine secreted by the pineal gland—elicits actin cytoskeleton rearrangements involved in early neuritogenesis [14,15]. In human olfactory neuronal precursors (ONPs), melatonin stimulates neurogenesis and acts as a neuro-differentiation factor that prompts the maturation and establishment of neuronal connectivity [16,17]. In newly formed neurons in the dentate gyrus and in already differentiated neurons of the hilar zone in the rodent hippocampus, melatonin induces dendritogenesis [18,19,20]. Moreover, we recently showed that melatonin increases the amount and velocity of extrasynaptic secretion from the soma and axons of cloned ONPs from a healthy subject [16], indicating that in addition to stimulating the structural connectivity of neurons the indoleamine also enhances their communication through secretion. The goal of this study was to evaluate the K^+^-evoked secretion of ONPs obtained from a patient with SCZ compared to secretion of cells from a healthy control subject (HCS), and to evaluate the effect of melatonin on this process and on the organization of actin microfilaments. Our main findings show that exocytosis is abnormally increased in SCZ-derived ONPs, and that melatonin treatment counteracts this augmented secretion.

## 2. Results

### 2.1. Characterization of Secretory Vesicles and of Potassium-Evoked Calcium Dependent Secretion in Human Olfactory Neuronal Precursors (ONPs)

#### 2.1.1. Vesicle-Associated Membrane Protein 1/2 (VAMP1/2)-Positive Secretory Vesicles are Present in Human ONPs

**Figure 1:**
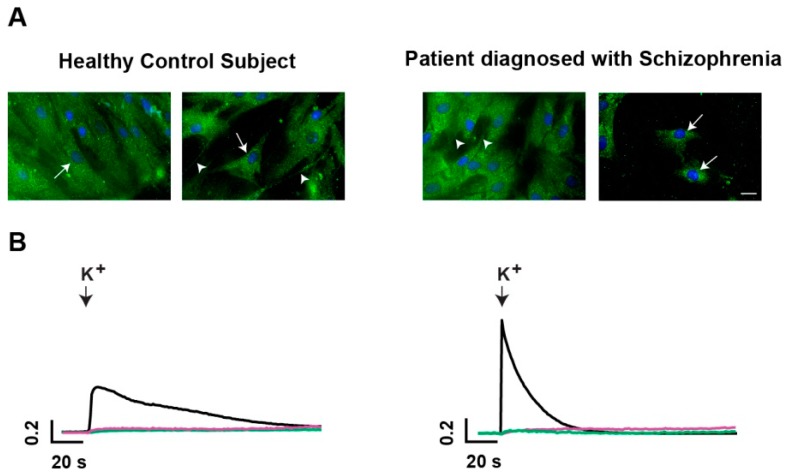
Characterization of secretory vesicles by VAMP1/2-immunostaining, and of K^+^-evoked calcium dependent secretion. Olfactory neuronal precursors (ONPs) obtained from healthy control subjects (HCS) and schizophrenia (SCZ) subjects were primary cultured up to passage 5 before immunostaining or evaluation of secretion. (**A**) Representative images of ONPs from HCS (**left**) and SCZ subjects (**right**), stained with a VAMP1/2 antibody, and followed by a fluorescein (FITC)-conjugated secondary antibody. Scale bar: 10 μm; (**B**) representative traces of the change in fluorescence of FM1-43, in response to depolarization with high potassium (K^+^) from HCS (**left**) and SCZ subjects (**right**). The time of depolarization is indicated by a black downward-pointing arrow. The increase in fluorescence reflects exocytosis. The pink traces represent the response of cells depolarized with high K^+^, in the presence of the calcium chelator EGTA, to prevent exocytosis. The green traces represent the response of cells mechanically stimulated by adding a physiological Hank’s solution.

To study secretion in ONPs, we first determined if neuronal precursors contain secretory vesicles by recognition of VAMP1/2 with a specific antibody. VAMP1/2 is a vesicle-associated membrane protein that participates in the fusion of vesicles with the plasma membrane during the process of exocytosis, and has been used as a marker of secretory vesicles. As shown in Figure 1A, VAMP1/2 was detected in the primary cultures of HCS- and SCZ-derived ONPs. The staining had a punctate pattern (Figure 1A), which is characteristic of vesicular structures [21]. In some cells, the VAMP-positive structures, which presumably correspond to vesicles or vesicle clusters, seemed to be widely distributed in the cytoplasm. In other cells, the VAMP-positive label appeared to be more densely distributed at the perinuclear zone (white arrows). Immunofluorescent structures were also observed in cell projections (white arrowheads).

#### 2.1.2. Characterization of Potassium-Evoked Secretion in ONPs

Once we determined the presence of secretory vesicles by VAMP1/2 immunolabeling, we depolarized the cells with high potassium (K^+^) (30 mM) and measured the increase in the fluorescence emitted by FM1-43 dye, to study the characteristics of secretion in these cells. FM1-43 is an amphiphilic compound that becomes fluorescent when inserted into the outer lipidic layer of the plasma membrane. Its fluorescence increases when secretory vesicles fuse with the plasma membrane, and more membrane surface is exposed to the FM1-43 dye [22]. Representative traces of the increase in the fluorescence of FM1-43 dye (plotted as dF/F as a function of time), before and after the depolarization with high K^+^, are shown in Figure 1B (black traces). This increase was not observed when only a mechanical stimulation was performed by adding physiological Hank’s solution (green traces); nor, when the cells were depolarized with high K^+^ in the presence of the calcium chelator EGTA in the extracellular medium (pink traces). The increase in fluorescence reflects exocytosis, which is a Ca^2+^-dependent process and requires a depolarizing stimulus. The depolarization-induced increase of fluorescence was transient, presumably due to photobleaching caused by repetitive illumination. Thus, we analyzed the maximum amplitude and the rising phase of the increase as indirect measurements of the amount and velocity of secretion, respectively. The slow and long-lasting kinetics of the increase in FM1-43 fluorescence suggested that exocytosis from ONPs is extrasynaptic. 

### 2.2. Comparison of Extrasynaptic Secretion from HCS- and SCZ-Derived ONPs and the Effects of Melatonin on Secretion

**Figure 2:**
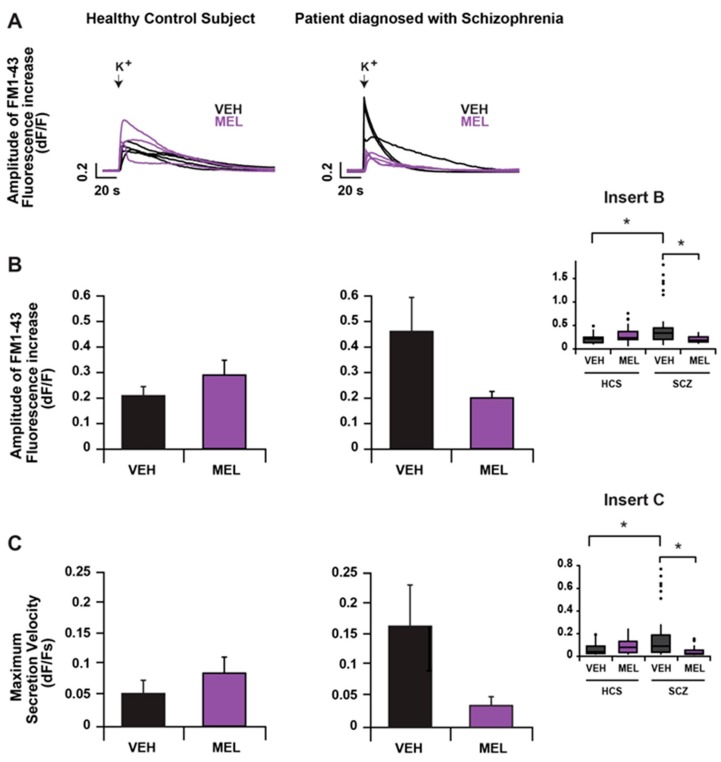
Secretion is increased in SCZ ONPs, and regulated by melatonin. FM1-43 fluorescence in ONPs obtained from a HCS (**left**) or SCZ (**right**), previously incubated with the vehicle (VEH; black) or with melatonin (MEL; purple), in response to depolarization with high potassium (K^+^). (**A**) Representative traces of the relative change in fluorescence of FM1-43 in ONPs. The time of depolarization is indicated by a black downward-pointing arrow. The increase in fluorescence reflects exocytosis; (**B**) maximum amplitude (mean + SEM; 27 cells from 9 experiments for each condition) of the increase in FM1-43 fluorescence, expressed as an increase over the basal fluorescence (dF/F); (**C**) maximum secretion velocity (mean + SEM; n as for the amplitude above), obtained from the maximum of the first derivative of the increase in fluorescence. Inserts in B and C panels show box plots of the corresponding data. Statistical analysis was done by one way analysis of variance (ANOVA) on ranks, followed by a post hoc Dunn's test with a Bonferroni adjust. Asterisks indicate significant differences (* *p* < 0.05).

We have previously shown that melatonin is an important factor that regulates neurodevelopment, and its deficit is probably associated with the etiology of schizophrenia. Therefore, we first studied whether secretion is altered in patient’s cells and then studied the effects of melatonin on extrasynaptic secretion. Figure 2A shows representative traces of the fluorescence kinetics (plotted as dF/F as a function of time), before and after the depolarization with high K^+^ in primary cultures of ONPs (obtained from a HCS (left) or from a SCZ subject (right), treated for 12 h with 10^−5^ M melatonin (MEL; purple traces) or with the vehicle (VEH; black traces)). Although the amplitude and kinetics of secretion varied between cells stimulated in the same conditions, it was clear that secretion in VEH-treated ONPs from the SCZ patient, had larger amplitudes and faster rising phases than ONPs from the HCS (Figure 2A; compare black traces from left and right panels). The increase in fluorescence had a total duration between 81 and 288 s, and variable amplitudes and kinetics. The slope of the rising phase of the fluorescence increase provides an indication of the velocity of exocytosis, and the time to the peak indicates the duration of this process, while the peak amplitude of the increase is an indirect measurement of the amount of fused vesicles, reflecting the amount of secretion. The fluorescence increases had peaks between 0.8% and 178% above the basal fluorescence level (0.008–1.788 dF/F), and rise times between 1 and 96 s, reflecting secretion with variable duration and velocity. Figure 2B,C shows bar plots of the mean and SEM of the amplitude (B) and the first derivative of FM1-43 fluorescence increase (C), reflecting the amount and velocity of secretion, respectively, in HCS (left) and SCZ (right), incubated with the VEH (black) or with MEL (purple). Since the data did not have a normal distribution, we used non-parametric statistics to compare these features between the groups. The inserts in Figure 2B,C show the box plots representing the 25th percentile, the median, and the 75th percentile of the amount and velocity of secretion, respectively. When the cells were treated with MEL, the HCS-derived ONPs showed a tendency to increase the amount and the velocity of secretion, but interestingly, MEL decreased these parameters in the SCZ-derived ONPs.

### 2.3. Evaluation of VAMP1/2-Immunofluorescence Intensity in HCS and SCZ-Derived ONPs

As a first approach to studying the mechanism by which secretion is increased in SCZ ONPs, and how MEL regulates secretion, we searched for a difference in the intensity of VAMP1/2 immunofluorescence as an indirect measurement of the number of secretory vesicles. Figure S1 shows box plots of this intensity in HCS and SCZ ONPs, incubated either with MEL or with the VEH. We did not find significant differences in the intensity of the VAMP1/2 immunofluorescent signal. The spatial distribution of the VAMP-positive label did not show any differences either. Thus, the marked increase in the amount and velocity of secretion observed in SCZ ONPs, with respect to the HCS ONPs and its regulation by MEL, were apparently not due to a different amount or distribution of secretory vesicles. Therefore, altered secretion in SCZ ONPs could be due to the process by which vesicles are mobilized and/or fused with the plasma membrane. In these processes, the cytoskeleton plays a key role in anchoring and mobilizing vesicles, and as a barrier to block vesicle fusion. Since our findings revealed that MEL regulates secretion (Figure 2), and it has been previously shown that the actin cytoskeleton is a target for MEL regulation [14,15], we searched for differences in the actin microfilaments of ONPs from SCZ, with respect to those of HCS.

### 2.4. Evaluation of Actin Microfilament Thickness in HCS- and SCZ-Derived ONPs

**Figure 3:**
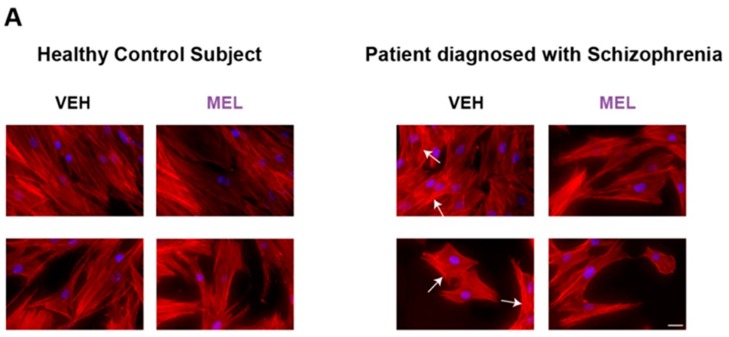

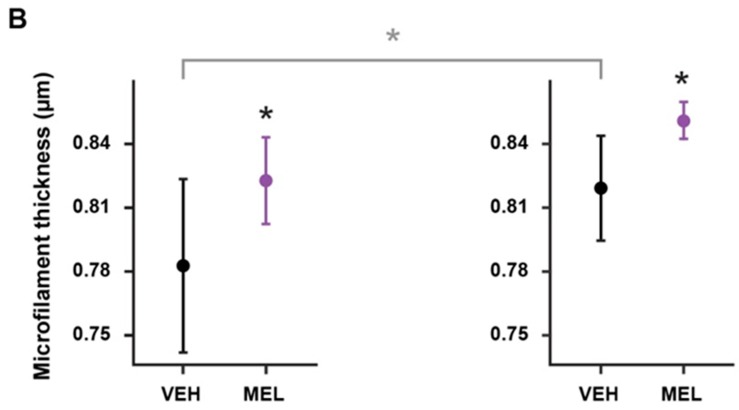
Melatonin increased the thickness of actin microfilament bundles in ONPs. ONPs obtained by exfoliation were cultured through passage 5 and incubated for 12 h, with either the vehicle (VEH) or 10^−5^ M melatonin (MEL). Cells were fixed and microfilaments stained with rhodamine (TRITC)-phalloidin. (**A**) Representative images of HCS- (**left**) or SCZ-derived ONPs (**right**), showing actin microfilament organization. Scale bar: 10 μm; (**B**) graph shows analysis of actin microfilament thickness (85 filaments/field from 10 fields for each condition). Results are expressed as the mean ± standard deviation. Black asterisks indicate significant differences (*p* < 0.05) of the MEL-incubated with respect to the VEH-incubated cells, determined by one way ANOVA and a Tukey post hoc test. The difference in the microfilament thickness between the VEH-incubated SCZ- and HCS-derived ONPs was also significant (gray asterisk).

Actin cytoskeleton was characterized by rhodamine (TRITC)-phalloidin staining in HCS- and SCZ-derived ONPs, incubated with either the VEH or 10^−5^ M MEL. This fluorescent toxin binds to F-actin with a high affinity [23]. As shown in Figure 3A, phalloidin(+)-filamentous structures that resemble actin microfilament bundles in a parallel arrangement were observed in both HCS- (left) and SCZ-derived (right) ONPs. Thicker actin microfilament bundles were evident in VEH-treated ONPs derived from the SCZ patient (Figure 3A, white arrows). Morphometric analysis confirmed that there are significantly thicker microfilaments in SCZ in comparison with HCS (*p* < 0.05). MEL treatment increased the microfilament thickness in both HCS- and SCZ-derived ONPs (Figure 3B).

## 3. Discussion

Alteration of neurotransmission systems in the brain has been suggested to underlie the pathophysiology of mental disorders. Herein, we showed that exocytosis is abnormally increased in neuronal precursors from a SCZ-patient. Also, our results indicated a modulatory effect of melatonin in this process, which might be mediated by its effects over the actin cytoskeleton.

Postmortem studies that measured the expression of constituent proteins of secretory granules and actin cytoskeletal proteins had suggested an altered secretion in SCZ [24]. However, direct measurements of neurotransmitter secretion from human CNS neurons have not been made, due to the low accessibility of neural tissue. In this regard, ONPs isolated from the human olfactory epithelium by a non-invasive method [25], provide a suitable preparation that mirrors both the neurodevelopment stages and the phenotypic alterations of neurons in neuropsychiatric diseases [26].

In schizophrenia, an augmented dopaminergic neurotransmission has been described, and therefore, the conventional antipsychotic treatment uses D2 antagonists to block the activity of these receptors. Studies in living patients have shown an exacerbated amphetamine-induced dopamine efflux in the striatum that correlates with an augmented displacement of a radioactive D3/D3-receptor ligand [27,28,29]. These studies strongly suggest that dopamine levels in the striatal presynaptic areas are increased. On the other hand, since occupancy of the D2/D3-receptor is higher, it is assumed that dopamine secretion is exacerbated. However, the mechanisms by which amphetamines induce dopamine release are independent of exocytotic pathways.

In this work we show for the first time that exocytotic mechanisms are altered in neuronal precursors from SCZ patients. Here, we evaluated K^+^-evoked secretion by the increase in the cumulative fluorescence intensity of a styryl dye. This method allows the study of the kinetics of exocytosis, which gives information about the mechanisms that produce and regulate this process. The amplitude of the response is an indirect measurement of the amount of vesicles that have fused to the plasmatic membrane and released their content to the extracellular milieu. The rate gives us information about the relative location of the vesicle pools at rest and their mobilization towards the plasma membrane in response to a stimulus. In addition, in polarized neurons, it allows the visualization of the cellular structures (somata, axons) from which exocytosis occurs. The time course of the exocytotic kinetics also allows us to distinguish whether secretion occurs from synaptic or extrasynaptic sites [12]. In this sense, we have previously described that human ONPs display an exocytotic pattern that resembles that of an extrasynaptic secretion [16].

Since the SCZ patient had not received any antipsychotic treatment at the time of the sample collection (i.e. näive for treatment), the increased secretion in SCZ-ONPs might correlate with the augmented dopaminergic tone observed in SCZ at the onset of the disease [10]. In agreement, an elevated amount of cathecolamines secreted from hiPSC-derived neurons of SCZ patients has been found before [11]. Nevertheless, a limitation of our methodology is that it does not provide information about the biochemical identity of the molecules that are released. In this regard, the complete secretome of human ONPs remains to be elucidated.

Melatonin treatment in SCZ-ONPs reduced the secretion rate and amplitude, and brought them to levels similar to those of HCS cells. In a previous study, we found that melatonin treatment increased the amplitude and velocity of secretion from a cloned culture of ONPs derived from a healthy subject [16]. In the primary-cultured HCS-ONPs studied here, we did not find a significant difference either in the amplitude or in the rate of secretion in the presence of melatonin. However, both parameters showed a tendency to increase when the cells were treated with the indoleamine. This apparent inconsistency could be explained because genetic homogeneity in the clone provides robustness of pharmacologic responses, while the more heterogeneous cell populations obtained in primary cultures increase the variability of the responses. The differential effect of melatonin in SCZ- and HCS-derived cells, i.e., a reduction or increase of secretion respectively, suggests that melatonin exerts a rheostatic modulation of the exocytotic process in ONPs. This kind of modulatory effect of melatonin has been described in other cellular systems, such as the immune system, and has even been proposed as a buffer-like effect [30]. In this regard it was reported that melatonin decreases the dopamine release in the retina, while no effects were elicited by the indoleamine in other brain regions, such as the striatum [31]. Moreover, even in different regions of the same brain structure, such as the ventral and dorsal hippocampus, there could be differential effects of melatonin on neurotransmitter secretion [32,33].

In our previous study, we also found that ONPs differentiation is impaired in SCZ [16]. Such impairment could lead to suboptimal connectivity for neurons which, in consequence, might have a deficient functionality in terms of the establishment of neural circuits. Melatonin treatment in these cells induced axogenesis in a dose-dependent manner, which strongly suggests that melatonergic signaling plays a key role in morphofunctional differentiation of neuronal precursors. It has been reported that SCZ patients display reduced melatonin serum levels [34]. In this sense, it is possible to infer that a melatonin deficiency could account for neuronal pathophysiology, including abnormal patterns of neurotransmitter secretion. Interestingly, our results indicate that neuronal morphofunctional impairment related to SCZ, might be counteracted by melatonin treatment. Thus, further clinical research would be necessary to assess melatonin potential as a therapeutic agent in SCZ, maybe in combination with current antipsychotic treatments. Also, since SCZ may have a neurodevelopmental etiology, an appropriate maternal melatonin supplement during pregnancy could be useful for the prevention of neural connectivity impairment associated with the disease. In terms of the cellular mechanisms that could account for an abnormal increase in exocytosis from SCZ-ONPs, several elements of the secretory pathway might be involved. To our knowledge, the presence of secretory vesicles in human ONPs had not been characterized before. Thus, our approach was to analyze VAMP1/2-immunoreactivity in these cells, to find out whether the altered exocytosis in SCZ-derived cells was correlated with changes in secretory vesicle density. VAMP proteins—also known as synaptobrevins—play a key role in the formation of the SNARE complexes that allow vesicle docking and fusion to the plasma membrane. Also, VAMPs seem to be essential for Ca^2+^-stimulated exocytosis, as shown previously [35,36]. Although our analysis did not reveal statistical differences in VAMP-immunofluorescence intensity, either between SCZ- and HCS-derived ONPs or melatonin- and vehicle-treated cells, we cannot discard subtle changes in VAMP1/2 expression in SCZ-derived cells. In this regard, studies reviewed by [24] suggest that the expression of synaptobrevins is not altered in SCZ. However, as these authors mention, there are only a few studies that have addressed this question. Expression of other secretory vesicle-associated membrane proteins, such as Ca^2+^-dependent activator protein for secretion 2 (CADPS2), was found increased in SCZ postmortem brain samples [37]. Thus, further characterization of constituent proteins of secretory vesicles in ONPs is needed to establish possible alterations in patients’ cells. On the other hand, as a possible underlying mechanism for striatal dopamine accumulation in SCZ patients, it has been proposed that more molecules of this neurotransmitter are packed within secretory vesicles [29]. Future studies in our laboratory including amperometric recording of human ONPs would address this question, and would also provide information about the biochemical identity of the neurotransmitters released.

In contrast with the intensity of VAMP-immunofluorescence, which does not seem to change in SCZ-cells, we show here that the actin cytoskeleton displays differences in SCZ-derived ONPs. In these cells, we found increased thickness of phalloidin(+)-filamentous structures that resemble bundles of actin filaments in a parallel arrangement. Intriguingly, melatonin treatment produced a thickening of actin microfilament bundles in both SCZ- and HCS-ONPs. Actin cytoskeleton has been previously proposed to be both a negative and a positive regulator of secretion [13]. Thus, in SCZ-cells which had an abnormally increased secretion, it is possible that thick actin microfilament bundles play a role as tracks to direct and facilitate the transport of the secretory vesicles to the fusion sites. However, further thickening of the actin cytoskeleton in melatonin-treated SCZ-cells could form a physical barrier, impeding the vesicle fusion with the plasma membrane. On the other hand, actin cytoskeleton may also act as a storage compartment for molecules that regulate exocytosis [38]. Among these molecules, the GTPase Cdc42 and the actin-related protein 2/3 (ARP2/3)-complex have been involved in actin cytoskeleton remodeling and regulated exocytosis from neuroendocrine cells [39]. Melatonin could influence the activation of these proteins [40]; however, the precise molecular mechanism by which melatonin modulates actin cytoskeleton dynamic rearrangements during exocytosis in ONPs remains to be studied.

In conclusion, our findings reveal that neuronal precursors from living SCZ-patients, which can be considered an ex vivo model of the disease, display an abnormally increased exocytosis that is returned to a normal level in the presence of melatonin. Differential effects in HCS- and SCZ-cells suggest that melatonin is a rheostatic hormone that restores the equilibrium in basic biological processes, among which is the secretion of biochemical mediators. Further research is needed to elucidate the mechanisms and signaling pathways by which melatonin modulates the exocytotic process in human neuronal precursors.

## 4. Materials and Methods 

### 4.1. Subjects

Participants were recruited from the Schizophrenia Clinic of the Instituto Nacional de Psiquiatría Ramón de la Fuente Muñiz at Mexico City. Diagnosis was established independently by two general psychiatrists, following the clinical criteria of the Diagnostic and Statistical Manual of Mental Disorders -fourth edition revised (DSM-IV R). All participants signed a written informed consent prior to their involvement in the study, which was previously approved by the Research Ethics Committee of the Instituto Nacional de Psiquiatría Ramón de la Fuente Muñiz (project number IC092010.0; January 2008–December 2017), in strict accordance to international standards. Olfactory neuroepithelial exfoliates were collected from a 28 year-old male patient diagnosed with paranoid SCZ, and from an age- and gender-matched healthy control subject (HCS) with no psychiatric precedents or medication. The SCZ participant was not receiving any medication at the time of the obtaining of the sample.

### 4.2. Primary Cell Culture

Olfactory Neuronal Precursors (ONPs) were collected by exfoliation of the anterior region of the medial lateral turbinate, as previously described [25]. Cells were cultured in Dulbecco’s Modified Eagle Medium/nutrient mixture F-12 (DMEM/F-12), supplemented with 10% heat-inactivated fetal bovine serum, 2 mM l-glutamine, 100 µg/mL streptomycin, 100 U/mL penicillin, and 250 ng/mL Fungizone™. Primary cultures were propagated and cryopreserved in an 8% dimethylsulfoxide (DMSO)-supplemented medium. The experiments were carried out with ONPs in passage 5. Cell culture reagents were obtained from Gibco^®^-Life Technologies (Grand Island, NY, USA). All other reagents were obtained from Sigma-Aldrich (St. Louis, MO, USA), unless otherwise stated. The acute effects of 10^−5^ M melatonin, added to the culture medium for 12 h before performing the experiments, were assessed.

### 4.3. Immunofluorescence

ONPs plated on glass coverslips were processed for immunofluorescence, as described in detail [25]. The secretory vesicle marker VAMP1/2 was immunodetected by overnight incubation with a 1:100 dilution of a specific monoclonal IgM antibody (raised in mouse (clone 3H3117)), which recognizes human VAMP1/2, followed by a secondary Fluorescein Isothiocyanate (FITC)-conjugated anti-mouse IgM antibody (raised in goat), diluted at 1:150 (both antibodies from Santa Cruz, Dallas, TX, USA; catalog numbers sc-73249 and sc-2082, respectively). Specificity of the primary antibody was reported by the supplier’s quality control department, as it recognizes the VAMP1/2 18 kDa-protein by Western blot in whole extracts of human-derived cell lines, and in mouse brain tissue extracts. As controls of our staining procedure, we omitted either the primary (anti-VAMP1/2) or the secondary (anti-mouse IgM) antibodies in parallel coverslips, which were instead incubated with 1% bovine serum albumin (BSA). These controls allowed us to test unspecific binding of the secondary antibody and autofluorescence, respectively. Nuclei were counterstained with 300 nM 4′,6′-Diamidino-2-phenylindole dihydrochloride (DAPI) (Molecular Probes, Eugene, OR, USA) for 10 min. All coverslips were mounted in PVA-DABCO medium. Preparations were observed with an epifluorescence, Nikon^®^ Eclipse TE2000inverted microscope, equipped with a DS-2MV Digital Sight camera (Minato, Tokyo, Japan), and acquired with the NIS-Elements 2.3 software (Nikon^®^, Melville, NY, USA).

VAMP1/2(+)-immunofluorescence (FITC channel) in the 800 × 600 px 32 bits images were deconvoluted with a theoretical PSF (point spread focal). All image analysis was done with the ImageJ software (version 1.51j8, U.S. National Institutes of Health, Bethesda, MD, USA) and the Iterative Deconvolve 3D plugin (DAMAS3, version 5.2, Bellevue, WA, USA). The PSF was generated with the Diffraction PSF 3D plugin (version 2.0, Bellevue, WA, USA). Images were then converted to 8 bits, background values were substracted, and VAMP1/2-fluorescence values were calculated as the ratio between the mean value of the FITC channel and the number of DAPI-stained nuclei in each field. 

### 4.4. Analysis of Secretion in Olfactory Neuronal Precursors

To study the secretory responses of the cultured ONPs and the effects of melatonin on this process, we measured the cumulative increase in the fluorescence of FM1-43, produced by the progressive exo/endocytosis of secretory vesicles [22]. This compound fluoresces only when bound to lipid membraneswhen it is present in the extracellular bathing solution. The fusion of secretory vesicles with the plasma membrane increases the membrane surface in contact with the dye, thus increasing its fluorescence. The increase in fluorescence was analyzed in the axons and somata of ONPs, obtained from a SCZ patient and from a HCS. To measure exocytosis, ONPs were plated on glass-bottomed culture dishes (NUNC^®^, Rosklide, Denmark) for 4 days. Twelve hours before evaluation of secretion, the culture medium was replaced by supplemented DMEM/F-12, containing 10^−5^ M melatonin or the vehicle (0.00001% ethanol), to assess the acute effects of melatonin. For exocytosis determinations, the culture medium was replaced by 1 mL physiological Hank´s solution (137 mM NaCl, 5.36 mM KCl, 1.26 mM CaCl_2_·2H_2_O, 1.09 mM MgCl_2_·6H_2_O, 0.81 mM MgSO_4_·7H_2_O, 4.2 mM NaHCO_3_, 0.44 mM KH_2_PO_4_, 1.33 mM Na_2_HPO_4_, 5.5 mM d-glucose), containing 2 µM of the fluorescent styryl dye FM1-43 (Molecular Probes, Eugene, OR, USA). Preparations were analyzed with the epifluorescence microscopy system described before, through a 40X oil-immersion objective (NA 1.30). Fluorescence measurements of FM1-43 were performed with an excitation filter passing 460–500 nm wavelengths and an emission (barrier) filter passing 510 nm. Image sequences of 640 × 480 pixels were acquired every 1 s for 300 s. After imaging for 120 s to measure basal fluorescence, neurons were depolarized by increasing the extracellular K^+^ concentration to 30 mM (high K^+^). This was achieved by adding 333 µL of modified Hank’s solution containing (22 mM NaCl, 120 mM KCl, 1.26 mM CaCl_2_·2H_2_O, 1.09 mM MgCl_2_·6H_2_O, 0.81 mM MgSO_4_·7H_2_O, 4.2 mM NaHCO_3_, 0.44 mM KH_2_PO_4_, 1.33 mM Na_2_HPO_4,_ 5.5 mM d-glucose, pH 7.4) to 1 mL of regular Hank’s solution (see above) in the dish. As a negative control, 333 µL of regular Hank’s solution was added to the culture dish instead of the high K^+^ solution. To verify that K^+^-evoked secretion was Ca^2+^-dependent, 200 nM of the calcium chelator ethylene glycol-bis(β-aminoethyl ether)-*N*,*N*,*N*′,*N*′-tetraacetic acid (EGTA) was added to the extracellular solutions (both in the dish and the depolarizing solution).

### 4.5. Actin Microfilaments Fluorescent Staining

ONPs plated on glass coverslips were fixed with 4% paraformaldehyde, during 10 min, and extensively washed with 0.01 M PBS pH 7.4. After membrane permeabilization with 0.1% Triton X-100 (15 min), cells were incubated with 200 nM Tetramethylrhodamine Isothiocyanate (TRITC)-phalloidin for 30 min at room temperature. Nuclei were counterstained with 300 nM DAPI. Preparations were observed with the epifluorescence microscopy system described before.

Phalloidin(+)-fluorescence (TRITC channel) 32 bit-images of 800 × 600 px size were deconvoluted with a theoretical PSF. After deconvolution, images were converted to 8 bits, background values were substracted, and final images were processed to binary (and then actin fibers were detected). Image analysis was performed with ImageJ 1.51j8 software, the Iterative Deconvolve 3D plugin (DAMAS3, v5.2), and the Diffraction PSF 3D plugin for PSF generation. Actin fibers in the TRITC channel were quantified and measured (length and width) with the Ridge Detection plugin (version 1.3, Max Planck Institute of Molecular Physiology, Dortmund, Germany). 

### 4.6. Statistical Analysis

Data were assessed for normality using Kolmogorov-Smirnov’s test. Secretion data were analyzed by one way analysis of variance (ANOVA) on ranks, followed by Dunn‘s post hoc test with a Bonferroni adjust [41,42]. Data from VAMP(+)-immunofluorescence and actin filament thickness was analyzed by one way ANOVA and a Tukey post hoc test. Differences between groups were considered significant when *p* < 0.05.

## Supplementary Materials

Supplementary materials can be found at www.mdpi.com/1422-0067/18/7/1439/s1.
