# Supplementary material for: Abnormally Increased Secretion in Olfactory Neuronal Precursors from a Case of Schizophrenia Is Modulated by Melatonin: A Pilot Study
Source: Int J Mol Sci. 2017 Jul 13;18(7):1439. doi: 10.3390/ijms18071439 (PMC5535930; doi:10.3390/ijms18071439)
Supplement: Supplementary file 1 [file ijms-18-01439-s001.pdf]

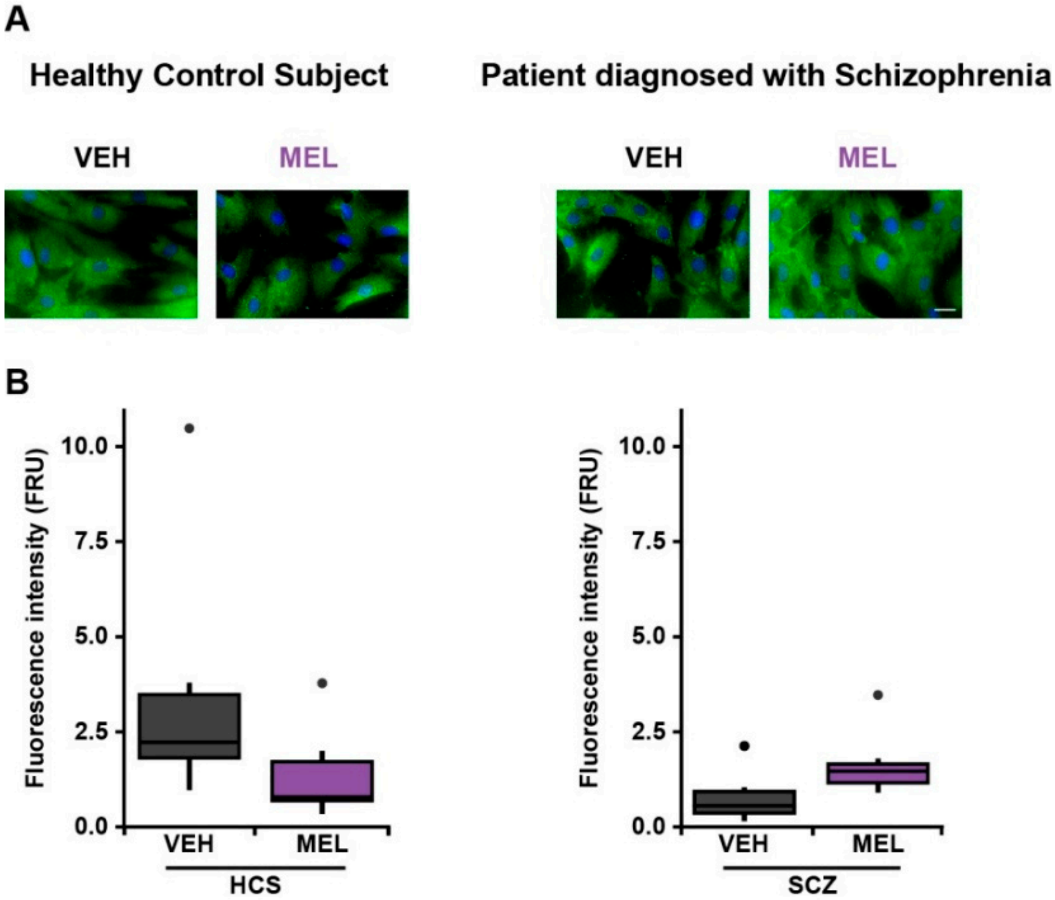

Figure S1. Evaluation of VAMP1/2-immunofluorescence intensity in ONPs obtained from HCS and SCZ subjects. ONPs were primary cultured up to passage 5 before immunostaining with a VAMP1/2 antibody followed by a FITC-conjugated secondary antibody. (**A)** Representative images of ONPs from HCS (**left**) and SCZ subjects (**right**) incubated with either the vehicle (VEH) or melatonin (MEL). Scale bar: 10 µm; (**B**) box plots of the corresponding data obtained from 6 visual fields for each condition; dots indicate the outliers. Statistical analysis was done by one way ANOVA. Differences between groups were not significant.
